# Mitochondrial augmentation of CD34^+^ cells from healthy donors and patients with mitochondrial DNA disorders confers functional benefit

**DOI:** 10.1038/s41536-021-00167-7

**Published:** 2021-09-24

**Authors:** Elad Jacoby, Moriya Ben Yakir-Blumkin, Shiri Blumenfeld-Kan, Yehuda Brody, Amilia Meir, Naomi Melamed-Book, Tina Napso, Gat Pozner, Esraa Saadi, Ayelet Shabtay-Orbach, Natalie Yivgi-Ohana, Noa Sher, Amos Toren

**Affiliations:** 1 Division of Pediatric Hematology and Oncology, Cell Therapy Center, The Edmond and Lily Safra Children’s Hospital, Sheba Medical Center, Tel Hashomer, Israel; 2 Sackler Faculty of Medicine, Tel Aviv University, Tel Aviv, Israel; 3 Minovia Therapeutics, Tirat Hacarmel, Israel; 4 Bio-Imaging Unit, Institute of Life Sciences, Hebrew University of Jerusalem, Jerusalem, Israel

**Keywords:** Haematopoietic stem cells, Cell biology

## Abstract

Mitochondria are cellular organelles critical for numerous cellular processes and harboring their own circular mitochondrial DNA (mtDNA). Most mtDNA associated disorders (either deletions, mutations, or depletion) lead to multisystemic disease, often severe at a young age, with no disease-modifying therapies. Mitochondria have a capacity to enter eukaryotic cells and to be transported between cells. We describe a method of ex vivo augmentation of hematopoietic stem and progenitor cells (HSPCs) with normal exogenous mitochondria, termed mitochondrial augmentation therapy (MAT). Here, we show that MAT is feasible and dose dependent, and improves mitochondrial content and oxygen consumption of healthy and diseased HSPCs. Ex vivo mitochondrial augmentation of HSPCs from a patient with a mtDNA disorder leads to superior human engraftment in a non-conditioned NSGS mouse model. Using a syngeneic mouse model of accumulating mitochondrial dysfunction (Polg), we show durable engraftment in non-conditioned animals, with in vivo transfer of mitochondria to recipient hematopoietic cells. Taken together, this study supports MAT as a potential disease-modifying therapy for mtDNA disorders.

## Introduction

Mitochondria are nearly ubiquitous cellular organelles that are involved in cellular processes including bioenergetics, homeostasis, metabolism, and apoptosis^1^. Mitochondria contain their own unique genetic coding material, a circular piece of mtDNA containing (in humans) 16,569 base pairs which encode for 37 genes^2^. Nearly every cell in the human body has multiple copies of mtDNA, ranging from hundreds to tens of thousands of copies^2^. Mitochondrial dysfunction related to mtDNA can be caused by pathogenic variants, including point mutations or deletions, or by mtDNA depletion (reduction in mtDNA copy number)^3^. Mutations or deletions in the mtDNA are associated with a heterogenous set of human diseases^4^ affecting numerous body systems^5^. To date, the only treatment available to patients with multisystemic mitochondrial disease is symptomatic, based on organ involvement and multidisciplinary surveillance; no therapy targeting disease causation exists^6^.

Preclinical in vitro and in vivo evidence supports physiological intercellular mitochondrial transfer as a source of cellular metabolic reprogramming, survival enhancement, and communication^7^. Early in vitro studies demonstrated that mitochondrial transfer between cells was capable of rescuing mitochondrial functionality^8^. Extensive in vitro evidence supports mitochondrial transfer between cells by mechanisms including direct intercellular transfer through tunneling nanotubes or through the release and subsequent internalization of vesicles through various endocytic routes (reviewed in ref. ^9^). In vivo mitochondrial transfer was first demonstrated in a mouse model of acute lung injury^10^, and has since been demonstrated between numerous cell types^11,12^. Data suggest that transfer of mitochondria, mtDNA or mitochondrial proteins is enhanced in cells undergoing metabolic stress, in pathological settings such as stroke^11,13^, epithelial injury^14^, Friedrich ataxia^15^, and cancer^16,17^. In addition, mitochondrial transfer between hematopoietic stem and progenitor cells (HSPCs) and bone marrow (BM) stromal cells has recently been demonstrated to be required for BM regeneration after conditioning^18^ and involved in the response to infection^19^.

In vitro uptake of purified mitochondria was shown to occur at low efficacy and was maintainable under selective pressure^20^. Subsequent studies demonstrated that injection of mitochondria to human cells could lead to rapid replacement of endogenous mtDNA, reconstituting recipient cells with exogenous normal mtDNA content^21,22^. Therefore, several methods are being developed to utilize mitochondrial transfer of exogenous mitochondria for therapeutic purposes^9,23–29^. Based on existing preclinical feasibility of in vitro mitochondrial uptake and in vivo intercellular mitochondrial transfer, we hypothesized that ex vivo supplementation of intact mitochondria is feasible and may lead to the metabolic rescue of recipient cells, which may propagate metabolic improvements via further intercellular in vivo mitochondrial transfer.

HSPCs are available recipient cells for mitochondrial transfer, accessible from the bone marrow, umbilical cord blood (UCB) or mobilized peripheral blood. Furthermore, allogeneic transplantation of healthy HSPCs has improved systemic pathologies in numerous non-hematopoietic tissues in preclinical models^30,31^. In a Friedrich ataxia mouse model, wild-type HSPC transplantation improved mitochondrial capacity in brain, skeletal muscle, and heart^15^, and HSPC-derived microglia and macrophages were demonstrated to transfer wild-type mitochondrial proteins to neurons and myocytes in vivo^15^. In a mouse model of a large-scale mtDNA deletion, HSPCs from bone marrow transplantation improved mouse survival and postponed renal failure by suppressing renal cell apoptosis^31^. Two different reports demonstrated improvement in hematopoietic and non-hematopoietic disease features in patients with Pearson syndrome (PS), a disease caused by a large-scale mtDNA deletion, following treatment with an allogeneic bone marrow or UCB transplantation^32,33^. Based on existing preclinical feasibility of in vitro mitochondrial uptake and in vivo intercellular mitochondrial transfer, we hypothesized that ex vivo supplementation of intact mitochondria to HSPCs is feasible and may lead to metabolic rescue of recipient cells, which may propagate metabolic improvements via further intercellular in vivo mitochondrial transfer.

In this study, we demonstrate the feasibility of enriching human HSPCs from healthy or diseased subjects with exogenous, functional mitochondria, a process we term mitochondrial augmentation. We further demonstrate long-term functional benefit of mitochondrial augmentation to patient-derived HSPCs administered to an immunocompromised murine model. Finally, we show that mitochondria from infused ex vivo augmented cells have a durable capacity to transfer to hematopoietic cells in the peripheral blood. Together, this study supports mitochondrial augmentation therapy (MAT) as a potential therapeutic avenue for patients with mtDNA deletions or mutations.

## Results

### Human HSPCs undergo mitochondrial augmentation in a dose-dependent manner

**Fig. 1:**
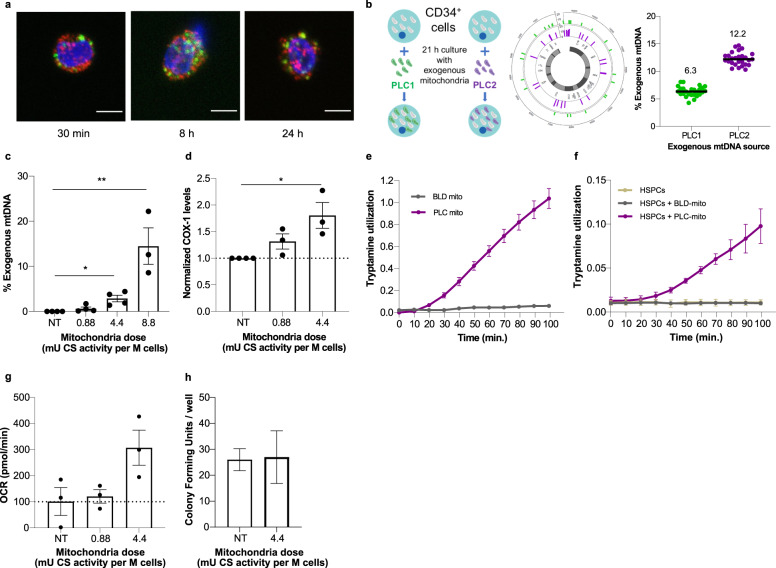
Mitochondrial augmentation occurs in human HSPCs and is a dose-dependent process. **a** Representative confocal microscopy images of human CD34^+^ stained with MitoTracker Orange and subsequently augmented with GFP-labeled mitochondria isolated from HeLa cells. Images were taken at 30 min, 8 h, and 24 h post augmentation; scale bar, 5 µm. Mitochondrial dose used was 4.4 mU CS activity per million cells. **b**–**d** Healthy donor CD34^+^ cells were augmented for 21 h with increasing doses of placenta-derived mitochondria. **b** Illustration of method of assessing percent of exogenous mtDNA content using NGS. Single nucleotide variants which differentiate between donor and recipient cell mtDNA are identified, and percent representation of donor mtDNA variants ((placenta-derived mitochondria batch 1 (PLC1) or 2 (PLC2)) in augmented cells are calculated. The average percent defined by all variants identified is reported as the percent exogenous mtDNA. **c** Exogenous mtDNA content quantified by NGS (average values ± SEM following augmentation with 0.88 mU CS (*n* = 4), 4.4 mU CS (*p = 0.0402 vs. NT cells, *n* = 4) or 8.8 mU CS (**p = 0.0020 vs. NT cells, *n* = 3) are presented (*p* values calculated by Kruskal–Wallis ANOVA followed by Dunn’s multiple comparison test, *p* = 0.0001 for the difference between three experimental groups (K–W stat = 13)). **d** COX-1 levels were determined using ELISA (0.88 mU CS, *n* = 3; 4.4 mU CS, *n* = 3, *p = 0.0199 vs. NT cells, calculated by Kruskal–Wallis ANOVA followed by Dunn’s multiple comparison test. *p* = 0.0071 for the difference between three experimental groups (K–W stat = 6.73). **e** The ability of blood (BLD) or placenta (PLC) derived mitochondria to utilize tryptamine was assessed using home-made tryptamine coated plates (*n* = 3 per BLD or PLC-derived mitochondria). **f** The ability of augmented or non-augmented healthy donor HPSCs to utilize tryptamine as a mitochondrial substrate was assessed using MitoPlate assay in HSPCs which were either non-augmented (*n* = 3), augmented with blood-derived mitochondria (HSPCs + BLD mito at 0.88 or 4.4 mU CS, *n* = 3), or augmented with placenta-derived mitochondria (HSPCs + PLC mito at 4.4 mU CS, *n* = 3). **g** Complex IV-dependent respiration in HSPCs augmented with placenta-derived mitochondria, 21 h post augmentation. Data presented as mean ± SEM. *n* = 3, *p* = 0.052 for 4.4 mU CS. **h** Healthy donor’s CD34^+^ cells were augmented with placenta-derived mitochondria. Ability to form colonies was tested 14 days (±2) post augmentation. n = 3 placental mitochondria batches. BLD blood, CS citrate synthase, HSPCs hematopoietic stem and progenitor cells, NGS next-generation sequencing, NT non-treated, OCR oxygen consumption rate, PLC placenta, PLC1 placenta-derived mitochondria batch 1, PLC2 placenta-derived mitochondria batch 2.

To assess the capacity of isolated mitochondria to enter HSPCs, the endogenous mitochondria of human CD34^+^ cells from a healthy donor were stained with MitoTracker Orange (MTO) and subsequently augmented with exogenous GFP-labeled mitochondria isolated from HeLa cells for up to 24 h. Using confocal microscopy, the presence of exogenous mitochondria in cells was visible as early as 30 min following augmentation and up to 24 h (Fig. 1a). Colocalization of endogenous (MTO labeled) and exogenous (GFP labeled) mitochondria was detected at the 24 h time point, consistent with fusion of exogenous mitochondria with the endogenous mitochondrial network (Supplementary Fig. 1). This confirmed feasibility of mitochondrial augmentation of human CD34^+^ HSPCs.

The human placenta is an excellent source of mitochondria, as it is a young, ephemeral tissue rich with healthy, functional mitochondria. To evaluate whether augmentation of HSPCs is a dose-dependent process, healthy human CD34^+^ cells were augmented with increasing doses of mitochondria isolated from human placenta. As exogenous mitochondria have haplogroup-specific single nucleotide polymorphisms (SNPs) which differentiate their mtDNA from the endogenous mtDNA of the recipient cells, this set-up provided an opportunity to study relative exogenous mitochondrial DNA (mtDNA) content post-mitochondrial augmentation using next-generation sequencing (NGS). For each augmentation process, all SNPs which differentiate between donor and recipient mtDNA were identified, the percent exogenous polymorphism present in mtDNA samples from augmented cells was analyzed per each SNP, and the average value reported (Fig. 1b and see “Methods” for details).

Mitochondrial content of isolated mitochondria was quantitated by their citrate synthase (CS) activity. Using different batches of donor mitochondria or recipient cells from healthy donors, increasing doses of mitochondria-to-cell ratios used for mitochondrial augmentation demonstrated a dose-dependent process, with levels of exogenous mtDNA ranging from 0.625 ± 0.37% in cells augmented with 0.88 mU CS activity per 1 × 10^6^ cells, up to 14.5 ± 4% exogenous mtDNA in cells augmented with 8.8 mU CS activity per 1 × 10^6^ cells (Fig. 1c). To confirm these NGS-based observations, we measured the protein level of mtDNA-encoded cytochrome c oxidase I (COX-1) in cells after mitochondrial augmentation. COX-1 levels, normalized to number of cells as quantitated by Janus Green staining, also showed a dose-dependent rise of 1.8 ± 0.24-fold with 4.4 mU CS per 1 × 10^6^ cells of mitochondria dose (Fig. 1d). A rise in relative COX-1 level per cell after mitochondrial augmentation may be due to one or more of the following reasons: (i) direct rise caused by entry of COX-1 protein present within the exogenous mitochondria, (ii) indirect rise due to new transcription and/or translation of COX-1 from internalized exogenous mtDNA, and (iii) indirect rise due to new transcription and/or translation of COX-1 induced by a cellular response to the mitochondrial augmentation process. While the latter options cannot be ruled out as at least part of the cause of the rise in COX-1 protein content after augmentation, together with NGS-based results and confocal imaging, mitochondrial augmentation was demonstrated to be a dose-dependent process that involves mitochondrial internalization into recipient HSPCs.

After mitochondria enter HSPCs during the mitochondrial augmentation process, enhanced mitochondrial activity is expected to be measurable as a result. Since healthy donor cells have normal levels of endogenous mitochondrial activity, we asked whether activity specific to placenta-derived exogenous mitochondria could be identified. To this end, to enable isolation of sufficient quantities of hematopoietic cell derived mitochondria, we used mitochondria from peripheral blood mononuclear cells (PBMCs) as a proxy for HSPC mitochondria. We then compared their activity to that of placenta-derived mitochondria using homemade tryptamine coated plates, based on the MitoPlate assay, which enables measurement of mitochondrial activity in response to metabolic substrates by measuring electron transfer. Only mitochondria derived from the human placenta (PLC), but not from peripheral blood (BLD), are able to utilize tryptamine as a substrate (Fig. 1e). Therefore, we hypothesized that if healthy donor HSPCs would be augmented with placenta-derived mitochondria, these should confer tryptamine-utilizing capabilities to recipient cells. While this enhanced metabolic activity is expected to be transient due to protein turnover in exogenous mitochondria, as the enzymes encoding tryptamine utilization are nuclear genome encoded, it is expected to be measurable at short time points after mitochondrial augmentation. As expected, only cells augmented with placenta-derived mitochondria, but not those augmented with PBMC-derived mitochondria or control non-augmented cells, could utilize tryptamine as a substrate for the electron transport chain (Fig. 1f). We also evaluated complex IV dependent oxygen consumption rate (OCR) of CD34^+^ cells augmented with placenta-derived mitochondria, 21 h post augmentation. We observed an increase of the OCR in a dose-dependent manner, rising by 20.4% in 0.88 mU CS dose augmented cells and by 207% for 4.4 mU CS dose augmented cells relative to non-augmented cells (Fig. 1g). This further demonstrates that mitochondria transferred during mitochondrial augmentation maintain functionality in recipient HSPCs. Finally, the in vitro ability of CD34^+^ cells to generate hematopoietic colonies did not change following augmentation with placenta-derived mitochondria (Fig. 1h, 4.4 mU CS/1 × 10^6^ cells dose).

### Patient-derived HSPCs also undergo mitochondrial augmentation

**Fig. 2:**
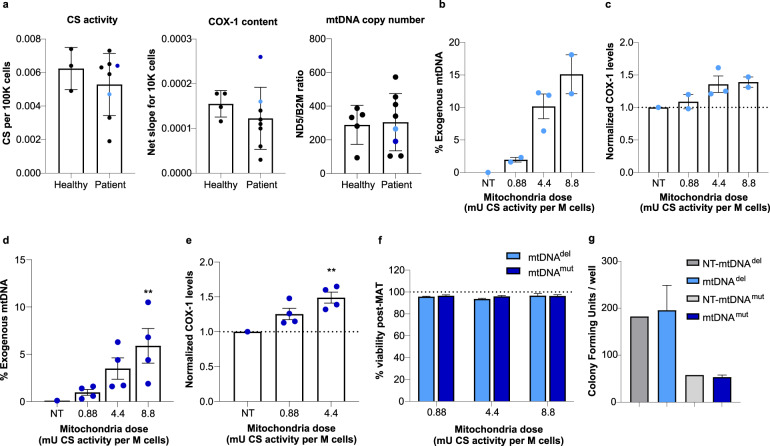
Mitochondrial augmentation of patient-derived HSPCs is a dose-dependent process and does not impair cell viability or functionality. **a** Mitochondrial activity or content of healthy and patient-derived HSPCs is variable, as quantitated by CS activity, COX-1 content, and mtDNA copy number. (*p* = 0.51, t(8) = 0.6837, *n* = 3 (healthy CD34^+^) and *n* = 8 (patient CD34^+^) for CS; *p* = 0.3995, t(10) = 0.8799 for COX-1 in unpaired *t*-test analysis, *n* = 4 (healthy CD34^+^) and *n* = 8 (patients’ CD34^+^). mtDNA copy number: *p* = 0.855, t(11) = 0.1864, *n* = 5 (healthy CD34^+^), *n* = 8 (patients’ CD34^+^) in unpaired *t*-test analysis. Light and dark blue dots represent the values for patient-derived samples used in experiments (**b**, **c**, light blue), and (**d**, **e**, dark blue). **b**, **c** mtDNA^del^ (Pearson Syndrome) patient CD34^+^ cells were augmented for 21 h with increasing doses of placenta-derived mitochondria. **b** Exogenous DNA analysis (*n* = 2 for 0.88 and 8.8 mU CS activity doses, *n* = 3 for 4.4 mU CS activity dose). **c** COX-1 analysis (single patient cells; *n* = 2 for 0.88 and 8.8 mU doses, *n* = 3 for 4.4 mU CS dose). **d**, **e** mtDNA^mut^ (Leigh Syndrome) patient CD34^+^ cells were augmented for 21 h with increasing doses of placenta-derived mitochondria batches. **d** Exogenous mtDNA content following mitochondrial augmentation were determined using NGS (single patient’s cells with *n* = 4 for each mitochondrial dose tested, *p* < 0.0001 for the difference between four experimental groups, Friedman test (*F*-stat = 12.00), **p = 0.003 for 8.8 mU CS activity dose, analyzed by Dunn’s pot hoc multiple comparison. **e** COX-1 levels were determined using ELISA (single patient’s cells with *n* = 4 for each mitochondrial dose tested, *p* = 0.0046 for the difference between four experimental groups, Friedman test (*F*-stat = 8.00) followed by Dunn’s post hoc test: **p = 0.0094 for 4.4 mU CS activity dose). **f** CD34^+^ cells from mtDNA^del^ (Pearson Syndrome) patient or mtDNA^mut^ (Leigh Syndrome) patient were augmented with increasing doses of placenta-derived mitochondria. Viability was assessed 21 h post augmentation using nucleocounter (mtDNA^del^ (single cell donor): 0.88, *n* = 2; 4.4, *n* = 3; 8.8, *n* = 2. MtDNA^mut^ (single cell donor): *n* = 4 for all doses). **g** CD34^+^ cells from mtDNA^del^ patient or mtDNA^mut^ patient were augmented with placenta-derived mitochondria. Ability to form colonies was tested 14 days (±2) post augmentation (mtDNA^del^ single cell donor; *n* = 3 placental mitochondria batches, *p* = 0.75, Wilcoxon matched-pairs signed rank test), mtDNA^mut^ (single cell donor): *n* = 4 placental mitochondria batches, ns vs. non-augmented cells, *p* = 0.625, Wilcoxon matched-pairs signed rank test). CS citrate synthase, HSPCs hematopoietic stem and progenitor cells, mtDNA mitochondrial DNA, NGS next-generation sequencing, NT non-treated.

HSPCs from patients with mitochondrial disease may not necessarily uptake isolated mitochondria to the same extent as those from healthy donors. While patient-derived samples were highly variable, patient-derived HSPCs did not significantly differ from those of healthy donor cells in their mitochondrial content as judged by CS activity, COX-1 content, and mtDNA copy number (Fig. 2a). To confirm the ability of mitochondria to augment HSPCs from patients with mtDNA deletion (mtDNA^del^) or mutation (mtDNA^mut^) syndromes, we repeated these experiments with CD34^+^ cells isolated from a patient with PS, a rare mitochondrial deletion syndrome, and Leigh syndrome (LS), caused in this patient by a mutation in the ND6 gene (14487 T>C). A similar dose-dependent augmentation of mtDNA^del^ HSPCs was seen, and the highest mitochondria to cell ratio (8.8 mU CS activity) assessed resulted in an average of 15.1 ± 3.0% exogenous mitochondrial DNA content in recipient cells (Fig. 2b), and an average of 1.39 ± 0.08-fold increase in COX-1 levels following mitochondrial augmentation (Fig. 2c). Similarly, mitochondrial augmentation of mtDNA^mut^ HSPCs resulted in an average of 5.9 ± 1.8% exogenous mitochondrial DNA content at the highest mitochondria to cell ratio (8.8 mU CS activity, Fig. 2d) and an average of 1.49 ± 0.08-fold increase in COX-1 levels (at 4.4 mU CS activity, Fig. 2e). Importantly, the mitochondrial augmentation process did not impair the viability of patient-derived CD34^+^ cells (mtDNA^del^ or mtDNA^mut^) in all dose levels assessed (Fig. 2f). In addition, functionality of CD34^+^ cells, as measured by their in vitro ability to generate hematopoietic colonies, remained intact following mitochondrial augmentation with placenta-derived mitochondria (Fig. 2g, 4.4 mU CS/1 × 10^6^ cells dose). Patient-derived CD34^+^ cells augmented with syngeneic blood-derived mitochondria (mitochondria isolated from maternal donor) further support CD34^+^ viability and functionality as assessed by in vitro colony-forming potential which is maintained after mitochondrial augmentation (Supplementary Figs. 2, 3, 0.88 mU CS/1 × 10^6^ cells dose).

Taken together, this data demonstrates that mitochondria isolated from blood or placenta can be used to ex vivo augment human CD34^+^ cells from healthy individuals or from patients with mtDNA^del^ or mtDNA^mut^, that transferred mitochondria used in augmentation are metabolically active after augmentation, and that this process does not compromise HSPC viability or ability to form colonies in vitro.

### Improved long-term engraftment of mitochondrially augmented patient-derived HSPCs

**Fig. 3:**
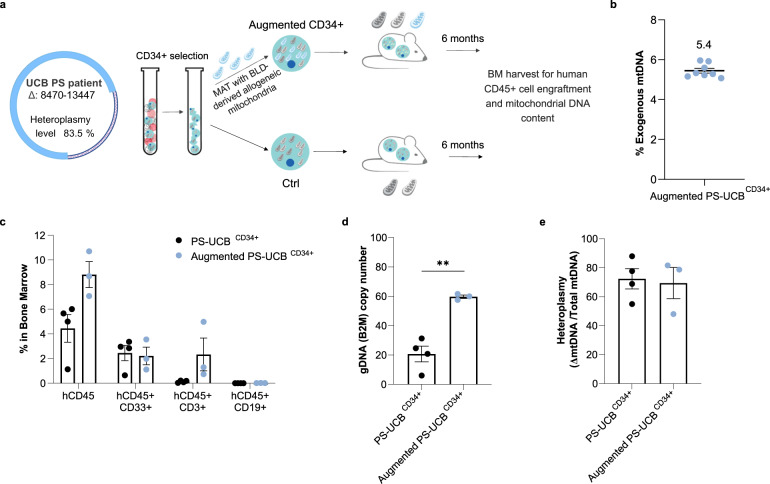
Mitochondrial augmentation of patient-derived HSPCs confers long-term engraftment benefit. **a** Graphical representation of experimental design. Limited PS-UCB derived CD34^+^ cell number enabled the treatment of *n* = 4 in control and *n* = 3 in mitochondrially treated groups. **b** Augmentation level of UCB-derived CD34^+^ cells, determined by percentage of exogenous mtDNA 21 h post augmentation assessed using NGS. **c** Level of human hematopoietic cells in NSGS mouse bone marrow, six months post transplantation measured by flow cytometry. *p* = 0.057 (U(4,3) = 0) for the differences in % hCD45^+^, hCD45^+^/CD33^+^ and hCD45^+^/CD19^+^ subpopulations in BM of mitochondrially-augmented vs. non-augmented mice groups. **d** Level of human cells in NSGS mouse bone marrow, analyzed by dPCR human B2M copy number assay **p = 0.0017. **e** Heteroplasmy levels of human mtDNA in NSGS mouse bone marrow six months post augmentation. BLD blood, dPCR Digital PCR, HSPCs hematopoietic stem and progenitor cells, MAT mitochondrial augmentation therapy, NGS next-generation sequencing, PS Pearson syndrome, PS-UCB Pearson syndrome umbilical cord blood, UCB umbilical cord blood.

To examine the long-term functional effect of mitochondrial augmentation in mtDNA^del^-derived HSPCs, we augmented UCB-derived CD34^+^ cells from a patient diagnosed with PS (PS-UCB^CD34+^) with blood-derived mitochondria (schematic in Fig. 3a). PS is a mtDNA deletion syndrome that usually presents in early life with bone marrow failure, leading to anemia and cytopenia in infancy^34^. This patient had a mitochondrial deletion spanning nt 8470 to nt 13,447, and heteroplasmy level of the cord blood unit was 83.5% as assessed by dPCR. We initially selected CD34^+^ cells from a thawed UCB unit and were able to harvest 1.2 × 10^6^ patient-derived CD34^+^ cells (PS-UCB^CD34+^). Mitochondrial augmentation was assessed by NGS as previously described and demonstrated 5.4 ± 0.12% exogenous mtDNA, 21 h post augmentation (Fig. 3b). Unconditioned NOD-SCID-Gamma-SGM3 (NSGS) recipient mice^35^ were intravenously injected with 5 × 10^4^ CD34^+^ cells (augmented and non-augmented). Six months after cell transplantation, mice were sacrificed, and engraftment of human hematopoietic cells in the bone marrow was assessed. Despite no conditioning, engraftment of human cells was evident in all animals. Using flow cytometry, we observed an increased number of human hematopoietic cells in the bone marrow of mice receiving augmented PS-UCB^CD34+^ cells in comparison with mice receiving non-augmented PS-UCB^CD34+^ cells (8.81 ± 1.05% of hCD45+ cells vs 4.45 ± 1.12%, Fig. 3c. For gating strategy see Supplementary Fig. 4). This was validated by dPCR quantification of human beta-2 microglobulin, designated to assess levels of human donor cells in the NSGS mouse (augmented PS-UCB^CD34+^ cells: 59.72 ± 1.2 vs. 20.7 ± 5.3 copies/ng DNA in the non-augmented PS-UCB^CD34+^, Fig. 3d). In a subset analysis, the main difference was due to an increase of hCD45^+^hCD3^+^ cells in recipients of augmented PS-UCB^CD34+^ cells (2.3 ± 1.3% vs. 0.13 ± 0.04% in augmented vs. non-augmented mice groups, respectively, Fig. 3c). Interestingly, heteroplasmy levels of human cells in the bone marrow did not change significantly (69.4 ± 10.7 vs. 72.4 ± 7.0 in augmented vs. non-augmented mice groups, respectively, Fig. 3e). Allogeneic blood-derived mitochondria were used as the mitochondria source in this experiment to enable tracking of mitochondrial persistence in vivo. Despite a long-term durability of the transplanted augmented PS-UCB^CD34+^ cells, a long-term persistence of exogenous mtDNA was not noted in this model 6 months post transplantation (data not shown). Taken together, this demonstrates that augmentation of mtDNA^del^ derived CD34^+^ cells enables higher human hematopoietic engraftment of diseased cells six months after treatment.

### Persistent in vivo mitochondrial transfer from mitochondrially augmented HSPCs in a diseased animal model

**Fig. 4:**
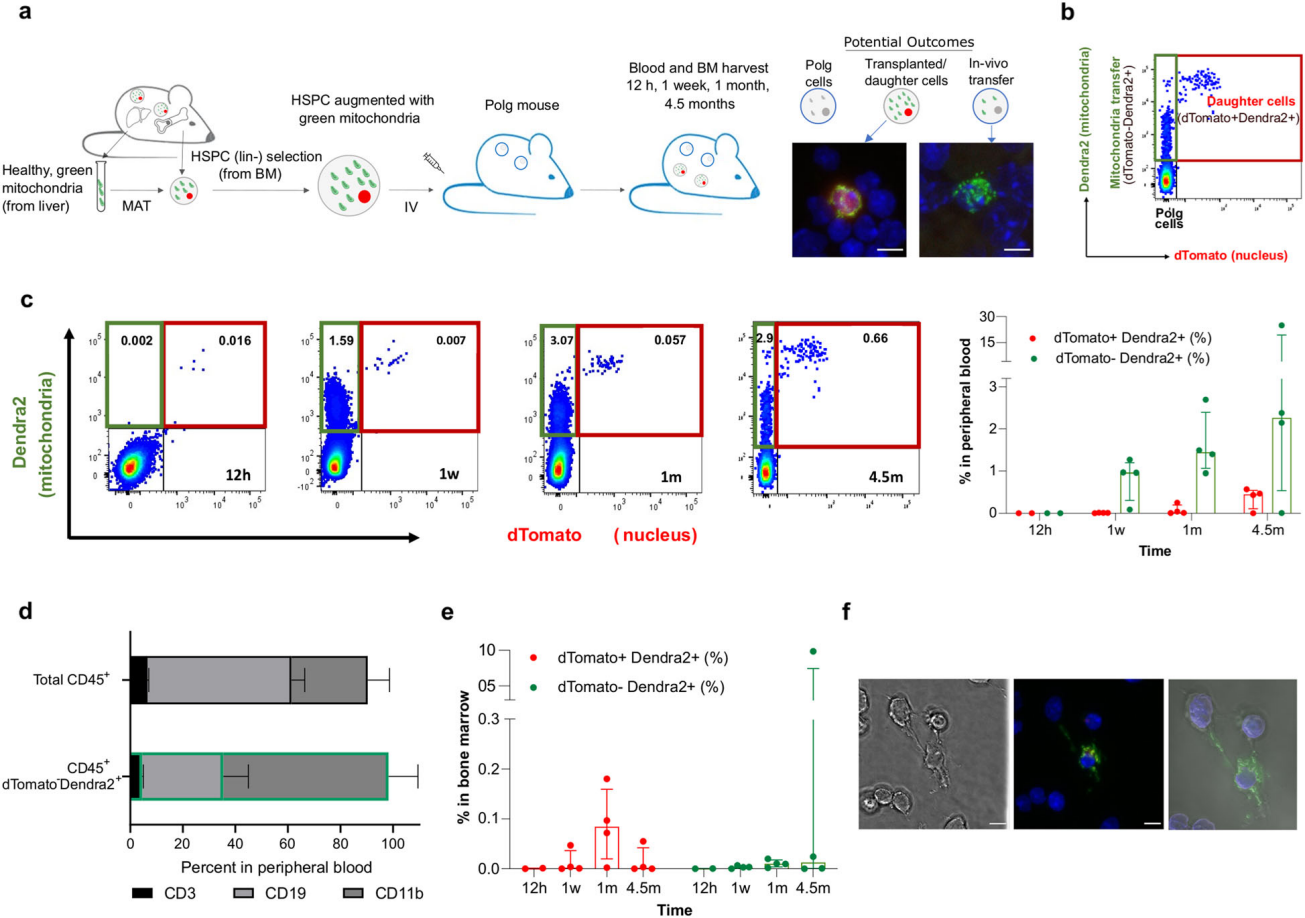
Demonstration of continuous transfer of exogenous mitochondria in blood of Polg mouse model. Lineage negative cells ubiquitously expressing red-labeled nucleus (dTomato) and green-labeled mitochondria (Dendra) derived from ROSA^nT-nG^/PhAM^excised^ mice were augmented with Dendra2^+^ mitochondria for 21 h and injected to Polg mice dosed at age 24–27 weeks (males) and 30-34 weeks (females). At 12 h, 1 w, 1 m, and 4.5 m post-injection blood was drawn and BM was extracted and both were analyzed by flow cytometry and confocal microscopy. **a** Graphical presentation of experimental design and a representative image of confocal microscopy of dTomato^+^Dendra2^+^ (left) and dTomato^-^Dendra2^+^ (right) found in bone marrow 1 m post-injection. Scale bar 5 µm. **b** Representation of flow cytometry analysis, which is expected to detect three types of cells: Polg cells, transplanted/daughter cells (red box, dTomato^+^Dendra2^+^) and Polg cells which received mitochondrial transfer (green box, dTomato^-^Dendra2^+^). **c** Representative figure of flow cytometry analysis of dTomato-Dendra2^+^ cells within CD45^+^ cell population in peripheral blood 12 h, 1 w, 1 m and 4.5 m post-injection (left). Percentages of dTomato^+^Dendra2^+^ cells and dTomato^-^Dendra2^+^ cells in peripheral blood (right). **d** Percentages of CD3^+^ T cells, CD19^+^ B cells and CD11b^+^ myeloid cells within the CD45^+^ and dTomato^-^Dendra2^+^ populations in peripheral blood 1 m post-treatment. **e** Percentages of dTomato^+^Dendra2^+^ cells (left) and dTomato^-^Dendra2^+^ cells (right) in the bone marrow. **f** Representative image of confocal microscopy analysis of BM extracted cells at 4.5 months after treatment, demonstrating Dendra2^+^ mitochondria transfer between adjacent cells. Left, differential interference contrast (DIC); center, merged florescent image from DAPI, Dendra2 and dTomato channels. Scale bar, 5 µm; right, 3D reconstruction of the Z-stack images. BM bone marrow, HSPCs hematopoietic stem and progenitor cells.

To assess the biodistribution of augmented HSPCs and to assess whether augmented HSPCs are capable of transferring mitochondria to other cells in vivo in a diseased animal model, we utilized genetically labeled cells injected into a mouse model of mitochondrial dysfunction. Specifically, two strains of mice were crossed, PhAM^excised^ strain (Jackson stock #018397) expressing mitochondria-specific green fluorescence (Cox8-Dendra2) and ROSA^nT-nG^ strain (Jackson stock #023537) expressing nuclear-specific red fluorescence (SRRM1-dTomato). Cells used for this study were harvested from ROSA^nT-nG^/ PhAM^excised^ compound heterozygous mice, in which nuclear dTomato and mitochondrial Dendra2 proteins are ubiquitously expressed. Bone marrow cells were harvested from mice, and lineage negative (Lin^-^) HSPCs enriched, as these are the murine equivalent of CD34^+^ selected human HSPC populations^36^. Lin^−^ HSPCs were augmented with Cox8-Dendra2^+^ mitochondria (isolated from livers of ROSA^nT-nG^/PhAM^excised^ compound heterozygous mice) for 21 h and injected to unconditioned Polg recipients (aged 6–8.5 months, see Fig. 4a schematic). Polg mice serve as a model of mitochondrial dysfunction and accelerated aging in which a Polg^D257A^ mutation impairs proofreading functions of the mitochondrial DNA polymerase-γ, leading to accumulation of mtDNA mutations and deletions^37^. At specific time points after mitochondrial augmentation therapy (12 h, 1 week, 1 month, and 4.5 months post-injection) animals were sacrificed, and cells from the bone marrow and peripheral blood were harvested and analyzed by confocal microscopy and flow cytometry based on their CD45^+^, dTomato and Dendra2 expression (Fig. 4a, b). In this experimental paradigm, three types of cells could be expected: (1) dTomato^+^Dendra2^+^ cells, the original cells infused or their daughter cells; (2) dTomato^-^Dendra2^-^, the recipient Polg cells; (3) Tomato^-^Dendra2^+^, cells which received in vivo mitochondrial transfer from the transplanted cells or from their progeny.

First, we assessed the engraftment of augmented cells, since Polg recipients were not preconditioned prior to cell injection. The percentage of dTomato^+^Dendra2^+^ cells in the peripheral blood increased over the time course from median of 0.0015% of the nucleated cells at 12 h post injection, to 0.45% of the nucleated cells 4.5 months after treatment (Fig. 4c), demonstrating engraftment and persistence of the cells. We next asked whether there was evidence for mitochondrial transfer, by assessing quantities of dTomato^-^Dendra2^+^ cells. The proportion of dTomato^-^Dendra2^+^ cells ranged from median 0.0015–2.26% of the nucleated cells in the peripheral blood of the mice (Fig. 4c). As the mitochondrial Cox8-Dendra2 protein is only encoded by the ROSA^nT-nG^/ PhAM^excised^ nuclear genome, and therefore its expression is transient once mitochondria are transferred to a Polg recipient cell, the consistent presence of dTomato^-^Dendra2^+^ cells in the peripheral blood is evidence of continual transfer of exogenous mitochondria which originated in infused augmented cells or their progeny. To quantitate the amount of transferred mitochondria in recipient cells, we utilized the MitoTracker Deep Red staining as well as inherent Dendra2 fluorescence of transferred mitochondria. We calculated that exogenous mitochondria were roughly 8.4% of total mitochondrial content in recipient cells (dTomato^-^Dendra2^+^) at 1 month and 6.1% at 4.5 months of the total cell mitochondria content (Supplementary Fig. 5). Importantly, this is a lower limit for exogenous mitochondria content, as it is limited by the half-life of the nuclear-encoded Cox8-Dendra2 protein which labels exogenous mitochondria.

The increase in dTomato^-^Dendra2^+^ cells suggests mitochondrial transfer occurs to hematopoietic cells residing in the peripheral blood. To identify which cells were the recipients of mitochondrial transfer, the distribution of the major subsets in peripheral blood was assessed in both total CD45^+^ and CD45^+^ dTomato^-^Dendra2^+^ populations (for gating strategy see Supplementary Fig. 6). Relative to the distribution of the hematopoietic subsets in the treated Polg animals (Fig. 4d, top bar), the subset distribution within cells containing exogenous mitochondria (CD45^+^ dTomato^-^Dendra2^+^ cells) shows that B, T and myeloid cells uptake exogenous mitochondria, and indicates that the myeloid cells, accounting for 61.85% of the CD45^+^ dTomato^-^Dendra2^+^ cells, preferentially uptake exogenous mitochondria.

Low levels of engraftment were expected in this model, as no preconditioning of the recipient Polg was performed prior to cell infusion. Indeed, the frequency of dTomato^+^Dendra2^+^ and dTomato^-^Dendra2^+^ cells within the CD45^+^ population is low in the bone marrow (Fig. 4e). However, the higher percentage of dTomato^+^Dendra2^+^ cells in the peripheral blood compared with that in the bone marrow is likely indicative of long-term persistence of daughter cells. Interestingly, confocal microscopy of a bone marrow sample extracted from a Polg recipient 4.5 m post-treatment shows a dTomato^-^Dendra2^+^ cell with Dendra2^+^ mitochondria located inside cytoplasmic nanotubes, potentially during mitochondrial transfer between a Dendra2^+^ cell and Polg cell (Fig. 4f).

## Discussion

In this study we demonstrated the feasibility of ex vivo mitochondrial augmentation of human peripheral blood derived CD34^+^ cells, human cord blood CD34^+^ cells and murine Lin^−^ bone marrow cells, without impacting HSPC cell viability or in vitro colony formation capacity. This proof of principle work further showed that mitochondrial augmentation of HSPCs is dose-dependent and confers functional benefit in vitro and in vivo in both healthy donor and patient-derived HSPCs. Finally, persistent mitochondrial transfer from infused augmented cells was demonstrated to multiple hematopoietic lineages in an animal model with mitochondrial dysfunction.

The ability of isolated mitochondria from various cell sources to enter numerous cell types in vitro, including a variety of cancer cells, healthy and patient-derived fibroblasts^25,38,39^, and immune cells^38^, is well documented. This process has been shown to be dose-dependent, and several studies demonstrated that exogenous mitochondria levels reach a peak beyond which they can be detrimental to cell viability or maximal mitochondrial function^40,41^. Dose-dependent entry of isolated mitochondria to a cancer cell line in vitro was quantitated to be 2–10% exogenous mitochondria as assessed by flow cytometry and up to 1% exogenous mtDNA as demonstrated by DNA sequencing^25^. Ex vivo mitochondrial enrichment has never been reported in HSPCs. Our studies demonstrate that HSPCs can be enriched with up to 22.2% exogenous mtDNA, which may arise from higher cell receptivity to exogenous mitochondria due to membrane permeability or composition^12,39^, differences in processing or culturing methodologies which enable higher mitochondrial uptake, or different mtDNA levels per mitochondrion from different cell sources^42–44^.

In vivo models of mtDNA deletion or mutations are rare. Thus, we utilized two different models to study in vivo properties of MAT, each with its advantages and disadvantages. To assess the potential effects of mitochondrial augmentation on human HSPCs, we used a humanized NSGS mouse transplanted with cord-blood derived CD34^+^ cells from a patient with PS, a mtDNA deletion syndrome. In this non-conditioned model, in which we did not expect complete human hematopoiesis, mitochondrially augmented CD34^+^ cells had improved long-term engraftment in the NSGS mice, as confirmed by flow cytometry and dPCR. Multi-lineage hematopoietic potential of engrafted cells was demonstrated, and animals that received mitochondrially enriched cells had significantly higher percentages of human CD3^+^ T cells. In the bone marrow, we could not detect donor mitochondria after 6 months, suggesting the effect of mitochondrial augmentation on functionality of HSPCs in this model either occurred early, or is independent of durable presence of exogenous allogeneic mitochondria.

Mitochondrial transfer occurs in vivo from stromal cells to HSPCs in response to infection^19^, to enable a rapid shift to oxidative phosphorylation to enable leukocyte expansion preceding to the transcriptional changes necessary to enable a cell autonomous rise in mitochondrial mass. It is tempting to speculate that mitochondrial augmentation of HSPCs mimics a physiological event to enable bioenergetic changes in HSPCs, which may in turn affect HSC fate, self-renewal, or regenerative potential^45,46^. These events may involve epigenetic mechanisms, such as those activated by the sirtuins^47,48^, which would not require long term persistence of exogenous mitochondria for durability of effect. The durable alterations which mitochondrial augmentation may have on HSPCs despite transient persistence of exogenous allogeneic mtDNA is an area of active investigation.

To investigate persistence in an immunocompetent animal, we used a syngeneic mouse model which enabled visualization of infused cells and their exogenous mitochondria. Recipient mice had a point mutation in the mitochondrial DNA polymerase gene, leading to accumulation of mtDNA mutations, representing a model for disease appropriate for such a study. This transplant model minimized rejection potential of exogenous mitochondria, aiming to enable maximal persistence of transferred cells and of exogenous mitochondria, both syngeneic to the recipient animal. We were able to not only show long term persistence of daughter cells, but we also observed continued in vivo transfer of exogenous mitochondria to recipient host hematopoietic cells, which continued to be present up to 4.5 months post transplantation. We showed that myeloid cells, as well as B cells, were the primary recipients of mitochondria in the peripheral blood. While in this model we did not assess the location in which mitochondrial transfer to peripheral blood cells occurred, recent evidence has suggested the presence of circulating functional mitochondria in the blood circulation^49^, and the capacity of hematopoietic cells to uptake functional exogenous mitochondria from isolated mitochondria as well as via cell-to-cell contact has been previously demonstrated^12,38^. Therefore, it is possible that durable exogenous mitochondrial transfer occurs either in the bone marrow or in the peripheral blood. This study supports two potential mechanisms of action of mitochondrial augmentation which are not mutually exclusive. Firstly, higher metabolic potential of HSPCs may lead to more balanced immune cell output. This is supported by improved engraftment and lymphoid output in our NSGS model and is consistent with a recent study that demonstrated that mitochondrial potentiation of HSCs ameliorated hematopoietic aging and ensured balanced lineage differentiation^50^. Secondly, as demonstrated by our study, exogenous mitochondria from augmented cells may be transferred to multiple immunological cell types, potentially providing systemic metabolic benefits^51^.

Mitochondrial metabolism is crucial for the function and inflammatory balance of immune cells^51–53^. Immune dysfunction is a major cause of morbidity and mortality in mitochondrial disease patients, which tend to have a higher risk of metabolic decompensations during infections, regardless of cytopenia, that may be due to cell intrinsic defects in immune cell mitochondrial function^52^. A growing body of preclinical evidence suggests that metabolic alterations in immune cell functionality may have systemic consequences for mitochondrial disease phenotypes^51,54^.

It remains to be determined whether exogenous mitochondria used during the augmentation process themselves persist in the syngeneic setting, or whether they exert durable metabolic effects on augmented HSPCs which are later translated to better metabolic functionality of daughter cells. Future studies aim to address this question as well as to further delineate in vivo alterations in immunophenotype and immune cell functionality after mitochondrial augmentation treatment of diseased animal models.

Systemic mitochondrial disorders are devastating rare diseases with no available therapy other than supportive care. Allogeneic stem cell transplantations may confer systemic benefit in hematopoietic and non-hematopoietic mitochondrial disease phenotypes in both clinical and nonclinical studies^31–33^ but are associated with morbidity and mortality and are not considered standard-of-care for any mtDNA disorder. Therefore, the possibility of treating mitochondrial disease symptoms by improving autologous HSPC functionality is tantalizing. As an autologous cell therapy in which functional mitochondria enrich patient-derived HSPCs, mitochondrial augmentation has the potential to alleviate symptoms of mitochondrial dysfunction in mtDNA disorders, without the risks associated with allogeneic HSCT. Taken together, these data provide evidence supporting feasibility of augmentation of human HPSCs and layout preclinical data for the potential of MAT as a therapeutic modality for mtDNA disorders.

## Methods

### Patient samples

A protocol for clinical trial (NCT03384420) and compassionate use program of autologous HSPCs augmented with maternal mitochondria was approved by Sheba Medical Center IRB and the Israeli Ministry of Health (MOH). The clinical trial was also conducted under IND #018343. Patients with mtDNA deletions or mutations were treated under this protocol, and informed consent was obtained accordingly from parents. Patient material was used under this protocol for clinical needs.

### Mitochondrial isolation and mitochondrial augmentation

Mitochondria were isolated from maternal peripheral blood mononuclear cells (PBMCs) or healthy donor term placenta using 250 mM sucrose buffer pH 7.4 by differential centrifugation. For mitochondrial augmentation, 0.88–8.8 mU of cryopreserved mitochondria (measured by CS activity, Sigma CS0720), were added to 1 × 10^6^ CD34^+^ cells suspended in 4.5% Human Serum Albumin solution. Cells and mitochondria were centrifuged at high speed (7000 × g, 5 min), then incubated for 21 h at room temperature.

### Cell imaging

Healthy donor CD34^+^ cells were stained with MitoTracker Orange (Invitrogen, California, US) and augmented with mitochondria isolated from HeLa cells expressing Turbo-green fluorescent (Turbo-GFP) mitochondria (CellTrend, Luckenwalde, Germany). At indicated times, cells were fixed for 10 min using 2% PFA (Electron Microscopy Sciences, Pennsylvania, US) and covered on slides using mounting solution containing 4′,6-diamidino-2-phenylindole (DAPI, SouthernBiotech, Alabama, US). Slides were visualized using confocal microscopy (FV1200, Olympus, Japan), equipped with a 60×/1.42 oil immersion. Images were analyzed using ImageJ software (National Institutes of Health, Bethesda, MD). The co-localization intensity profile was generated with an ImageJ macro measuring the intensity profile of the different channels across a line.

### Next-generation sequencing (NGS)

After augmentation, CD34^+^ cells were washed two or three times, and DNA was isolated from 40,000 cells using QIAmp DNA micro kit (Cat# 56304 Qiagen, Hilden, Germany) according to the manufacturer’s instructions. Mitochondrial DNA was amplified by two alternative methodologies, PCR-based and RCA-based, as detailed below, with comparable results in % exogenous mtDNA (see Supplementary Fig. 7a for comparison between results from both methodologies). As PCR-based methods are prone to biases (Supplementary Fig. 7b), RCA-based mtDNA amplification prior to NGS sequencing was used in most analyses.

### Two primer set PCR-based method

DNA was processed at the Research Resources Center Genome Research Core (University of Illinois, Chicago). Mitochondrial DNA (mtDNA) was amplified following the Illumina Human mtDNA genome guide^55^. Briefly, 1 ng purified human DNA was amplified in a 50 µl reaction containing 2.5 U of TaKaRa LA Taq and either primer pair MTL-F1 and MTL-R1 or MTL-F2 and MTL-R2. MTL1-F (5′-AAAGCACATACCAAGGCCAC), MTL1-R1 (5′-TTGGCTCTCCTTGCAAAGTT), MTL-F2 (5′-TATCCGCCATCCCATACATT), MTL-R2 (5′- AATGTTGAGCCGTAGATGCC). Cycling was 94 °C for 15 s and then 30 cycles of: 98 °C for 15 s, 68 °C for 10 s (slow ramp from 68 °C to 60 °C at 0.2 °C per second), 60 °C for 15 s, 68 °C for 11 min, followed by a 72 °C hold for 10 min then hold at 10 °C. Resulting amplicons were analyzed by Agilent Tapestation on genomic DNA tape. Amplicon concentrations were normalized and pooled by sample at 20 ng/µl and used without any additional cleaning with 100 ng pooled amplicons as input to the Illumina Nextera FLEX library protocol. Indexing was performed with Illumina UDI indexes with 5 cycles of PCR. Resulting libraries were pooled and sequenced on an Illumina MiniSeq MO sequencing flow cell at 2 × 150 bp, with a minimal coverage of 2500×.

### Rolling cycle amplification (RCA)

Total DNA was processed with REPLI-g mitochondrial DNA kit (QIAGEN, CAT#151023) according to the manufacturer’s instructions from roughly 10 ng DNA. Resulting material was cleaned with 0.5× ratio SPRI Beads (AMPure XP, Beckman Coulter) analyzed by Agilent Tapestation on genomic DNA tape. 100 ng input DNA was sheared using Covaris E220 Focused-ultrasonicator (Covaris) with the following 350 bp insert setting: duty factor 10%, peak power 175 W, cycles/burst 200, duration: 50 s, temperature 5–10 ºC. Sequencing libraries were prepared using TruSeq DNA Nano Library Prep Kit (Illumina) according to the manufacturer’s protocol. Libraries were sequenced on Illumina Miseq sequencer with 150 bp paired ends read length, with a minimal coverage of 2500× for mitochondrial sequence.

### NGS Data analysis

Raw reads were trimmed by Trim Galore (Babraham Bioinformatics). Trimmed data were aligned against the mtDNA rCRS reference sequence (NC_012920.1) with the Burrows-Wheeler Aligner (BWA-MEM version 0.7.17) using default parameters. Variant calling comparing exogenous and endogenous (using Basic Variant Detection 2.0 algorithm within CLC genomics for samples processed using two primer PCR based method by Research Resources Center Genome Research Core (University of Illinois, Chicago) core, or by Mutserve seppinho (GitHub) within mtDNA server. Data were combined by Python script and Informative SNPs, defined as variants that differentiate between exogenous and endogenous mtDNA were identified. The percent exogenous mtDNA was calculated by retrieving the SNP frequency from the bam files for each informative SNP per sample.

### COX-1 quantification

After mitochondrial augmentation, cells were collected and washed twice with PBS. Mitobiogenesis in-Cell ELISA kit (Abcam., Cambridge, UK cat#ab110217) was used to assess protein levels of mitochondrially encoded cytochrome c oxidase subunit 1 (COX-1), normalized to Janus green staining quantification of total cell number, using infinite pro 200 plate reader (TECAN, Männedorf, Switzerland).

### Mitochondrial activity

Mitochondrial activity within CD34^+^ cells was measured using the MitoPlate S-1 (Biolog, California, US) following the manufacturer’s instructions. Briefly, plate substrates were fully dissolved using an assay mix containing Biolog MAS (Biolog, California, US), redox dye MC (Biolog, California, US) and 45 µg/ml saponin (Sigma-Aldrich, Missouri, US) for 1 h at 37 °C. CD34^+^ cells were washed with twice with PBS, resuspended in MAS at a density of 6 × 10^4^ cells/30 µL and added to wells. Kinetic reading at 590 nm was done using infinite pro 200 plate reader (TECAN, Männedorf, Switzerland). Background readings (no substrate) were subtracted from absorbance readings.

Activity of isolated mitochondria was measured using empty plates (Biolog, California, US) coated with 10 mM tryptamine (Sigma-Aldrich, Missouri, US). 40 µg of isolated mitochondria was resuspended in Biolog MAS. Kinetic reading at 590 nm was done using infinite pro 200 plate reader (TECAN, Männedorf, Switzerland). Background readings (no substrate) were subtracted from absorbance readings.

### Oxygen consumption rate

CD34^+^ cells augmented with placenta-derived mitochondria were analyzed for mitochondrial function using Seahorse XFe96 Analyzer (Agilent Technologies, CA, USA). Oxygen consumption rate (OCR) was measured using specific substrates for complex IV (CIV). Following augmentation, cells were treated with 0.2 mg/ml saponin (Sigma-Aldrich) and were maintained in RPMI medium (Agilent Technologies, CA, USA) supplemented with glucose (10 mM), pyruvate (1 mM) and glutamate (2 mM) (all from Agilent technologies). Pre-coated Seahorse XF96 microplate (Cell-tak, Corning, NY, USA) was loaded with 50 µl medium containing 300,000 cells. To attach cells, plate was centrifuged at 700 × *g* for 1 min with no break, rotated 180 °C and centrifuged again at 700 × *g* for 1 min with no break. The plate was incubated in 37 °C in a non-CO_2_ incubator for 30 min followed by adding 130 µl of RPMI medium to each well. For CIV analysis, ADP was injected to port A (5 mM, final concentration), rotenone and antimycin A to port B (0.5 µM/2.5 µM), TMPD (N,N,N′,N′-tetramethyl-p-phenylenediamine) and ascorbate (ASC) to port C (500 µM/2 mM) and sodium azide (100 mM) to port D. Mix and measure times were 3 min each. Wave software (Agilent technologies) was used to analyze OCR rates. Complex IV-dependent respiration was calculated by subtracting OCR (TMPD/ASC) values from those of OCR inhibitor (sodium azide).

### Citrate synthase (CS) activity assay

Basal CS activity in healthy and patient CD34^+^ cells were determined using CS activity assay kit (Sigma, #MAK193). Colorimetric product proportional to enzymatic activity was determined using infinite pro 200 plate reader (TECAN, Männedorf, Switzerland).

### Cell viability

Viability of CD34^+^ cells was assessed after augmentation using the automated cell counter Nucleocounter NC-200 (Chemometec, Allerod, Denmark).

### Colony-forming assay

Augmented vs. non-augmented CD34^+^ cells (5 × 10^3^ cells/ml in MethoCult medium) were plated onto a meniscus free SmartDish (STEMCELL Technologies, Vancouver, Canada) and incubated at 37 °C, 5% CO_2_, as per the manufacturer’s instructions. Following 14 ± 2 days, the culture plate was placed top of a STEMgrid (STEMCELL Technologies, Vancouver, Canada) and colonies were manually counted using a standard light microscope.

### Digital PCR (dPCR)

Murine bone marrow DNA was isolated using QIAamp DNA Micro kit (Qiagen, Hilden, Germany) and quantified with Qubit dsDNA High Sensitivity kit (Invitrogen, California, US). 2 ng (for B2M) or 6.55 ng (for total and full-length mtDNA analysis) of total DNA template were mixed with QuantStudio 3D digital PCR Master Mix and loaded onto digital chips [QuantStudioTM 3D Digital PCR 20K Chip kit v2 and Master Mix (Applied Biosystems)] and thermally cycled in a ProFlex Flat PCR System (ThermoFischer, 96 °C for 10 min followed by 39 cycles (for B2M) or 45 cycles (for total mtDNA copy number determination by A16162G and A10831G mitochondrial assays, or full-length) of 60 °C for 2 min and 98 °C for 30 s, and 60 °C for 2 min). Digital chips were then read in QuantStudio 3D dPCR analyzer (ThermoFischer) and analyzed in Quantstudio 3D AnalysisSuite software. Copy numbers were adjusted to ng DNA. B2M, total and full-length mtDNA copy numbers used for UCB unit heteroplasmy determination (full-length 9254G and total mtDNA A16162G assays) or CD34^+^ heteroplasmy determination (full-length A10831G and total mtDNA A16162G assays) were determined by dPCR with the following assay sequences. Copy numbers of total and full-length UCB mtDNA were used for heteroplasmy level determination.

**Table:** 

B2M assay	Probe: FAM/ATGTGTCTG/ZEN/GGTTTCATCCATCCGACA
	F Primer: CCAGCAGAGAATGGAAAGTCAA
	R Primer: TCTCTCTCCATTCTTCAGTAAGTCAACT
A16162G SNP Assay	Probe A allele: FAM/AGTACATAAAAACCC
	Probe G allele: VIC/CATGAAAACCCA
	Primer F: CTGCCAGCCACCATGAATA
	Primer R: GGTTGATTGCTGTACTTGCTTG
A10831G SNP Assay	Probe A allele: VIC/TTGAATCAACACAACC
	Probe G allele: FAM/ TTGGATCAACAC
	Primer F: CTACCACTGACATGACTTTCC
	Primer R: GTAGAGGGATGATGCTAATAATTAGG
9254G SNP Assay	Probe G allele: FAM/AT+G+G+CC+CCT+AA
	Primer F: CCTGCACGACAACACATAA
	Primer R: GTCATTAGGAGGGCTGAGA

### Animal studies—NSGS

Animal care was in compliance with protocols approved by the Council for Animal Experiments, State of Israel Ministry of Health, in conformity with the Animal Welfare Law guidelines. All animal experiments were approved by Sheba Medical Center institutional ethical board for animal experiments (protocols 1095/17/ANIM and 1252/20/ANIM). Female NSG-SGM3 (NSGS) mice were purchased from Jackson laboratories. All mice were housed at a temperature of 25 °C under a 12-h dark/light cycle.

CD34^+^ cells were isolated from an umbilical cord blood (UCB) unit of a mtDNA^del^ patient using CD34^+^ separation column according to manufacturer’s instructions (130-090-101, Miltenyi biotech), and either augmented with human blood-derived mitochondria at 0.88 mU CS dose, or incubated without mitochondrial augmentation. Following 21 h, augmented and non-augmented cells were washed, resuspended in 4.5% HSA solution and intravenously injected to the tail vein of NSGS mice (aged 3 weeks). Mice were administered with control (non-augmented, *n* = 4) or augmented (*n* = 3) CD34^+^ cells (50,000 cells/animal).

### Flow cytometric analysis—NSGS mice

For flow cytometry analysis of bone marrow cells, NSGS mice were euthanized 6 months post-injection. Isolated bone marrow cells were incubated for 5 min with RBC lysis buffer (Biolegend, California, USA), washed with PBS and stained for 30 min with different combinations of the following fluorochrome-conjugated antibodies: hCD45-FITC (Cat. 304006, dilution 1:20), mCD45-APC/Fire750 (Cat. 103154, dilution 1:50), CD33-PE (Cat. 366608, dilution 1:50), CD3-APC (Cat. 300412, dilution 1:66), CD19-PerCP (Cat. 363014, dilution 1:20). All antibodies were purchased from Biolegend (Biolegend, California, USA). Cells were then washed with PBS, and resuspended in FACS buffer (PBS with 2.5% FBS) prior to acquisition on Gallios (BD Biosciences, California, USA) and data analysis with FlowJo Software (Treestar, Oregon, USA). For compensation, BD CompBeads (Cat.No. BD552843 and BD552845) were used according to the manufacturer’s instructions.

### Animal studies—Polg

All animals were cared for in compliance with protocols approved by the Council for Animal Experiments, State of Israel Ministry of Health, in conformity with the Animal Welfare Law guidelines. All experimental procedures were carried out in accordance with the ethics commission with an approval number IL-19-3-129. All mice were housed at a temperature of 25 °C under a 12h dark/light cycle in Envigo CRS (Israel).

Cell donor mice were compound heterozygous ROSA^nT-nG^/ PhAM^excised^ mice purchased from Jackson Laboratories, created by crossing PhAM^excised^ mice (Stock #: 018397) with ROSA^nT-nG^ mice (Stock #023537). Recipient mice were the Polg mouse model (Jax stock# 017341). Lin^-^ cells were isolated from a ROSA^nT-nG^/ PhAM^excised^ bone marrow cell suspension using a Lin^-^ separation kit (EasySep, cat# 19856, STEMCELL Technologies, Vancouver, Canada). These were augmented with mitochondria derived from livers of ROSA^nT-nG^/ PhAM^excised^ mice. Polg male mice (*n* = 8) were injected with 124,000 augmented cells (derived from male ROSA^nT-nG^/ PhAM^excised^ mice); Polg female mice (*n* = 10) were injected with 126,000 augmented cells (derived from female ROSA^nT-nG^/ PhAM^excised^ mice).

### Flow cytometric analysis—Polg mice

For flow cytometry analysis of peripheral blood and bone marrow cells, Polg mice were euthanized at 12 h, 1 w, 1 m, and 4.5 m post-injection. Isolated bone marrow cells were stained for 25 min with different combinations of the following fluorochrome-conjugated antibodies: CD45-Pacific blue (Cat. 103126, dilution 1:50), CD11b-PerCP (Cat. 101229, dilution 1:50), CD19-PerCP (Cat. 115531, dilution 1:50), CD3-PE/Cy7 (Cat. 100219, dilution 1:50). All antibodies were purchased from Biolegend (Biolegend, California, US). Cells were then washed with PBS, fixed with 1% PFA (Electron Microscopy Sciences, Pennsylvania, US) for 10 min at 4 °C and resuspended in FACS buffer (Biolegend, California, USA) prior to acquisition on BD LSRFortessa (BD Biosciences, California, USA) and data analysis with FlowJo Software (Treestar, Oregon, USA). Blood samples were lysed twice with Red Blood Cell (RBC) lysis buffer prior to antibody staining as described above.

At 1 m and 4.5 m post injection cells were stained for 30 min at 37 °C with MitoTracker Deep Red (ThermoFisher scientific), washed with PBS, fixed with 1% PFA for 10 min at 4 °C and resuspended in FACS buffer prior to analysis.

### Imaging of single cell suspensions

Peripheral blood cells were washed with 1× Red Blood Cell (RBC) Lysis Buffer (BioLegend Cat. No. 420301). Bone marrow cells were from femur and tibia in PBS. Cells were centrifuged directly to the slide using Cellspin (THARMAC, Wiesbaden, Germany) (800 rpm, 3 min), fixed with 1% PFA (Electron Microscopy Sciences, Pennsylvania, US) for 10 min, stained with Fluoro-GelII mounting medium containing DAPI (Bar-Naor BN17985-50), covered with cover slip and sealed. Images were visualized using Zeiss LSM780 Inverted Confocal Microscope (Plan-Apochromat 63 × 1.4 N.A. oil immersion objective). Cells were scanned with 561 nm for dTomato, 488 nm for Dendra2 and then with 405 nm for DAPI and transmitted light image.

### Histological slides

Samples were harvested and fixed in 2.5% PFA for 48 h. Prior to dissection samples were put in 30% sucrose as cryoprotectant for 48 h. Then, organs were trimmed in −20 °C using a cryostat in a standard position per organ and put in an embedding OCT compound cassette. OCT blocks were sectioned at ~12 microns thickness, fixed again in 4% PFA for additional hour, slides were mounted by fluorescent mounting medium with DAPI and put on a glass slide. Several slides were stained with CD45 antibody prior to mounting. For immuno-fluorescence assays, the fixed sections were blocked with 10% goat serum for 1 h at room temperature (RT). Primary Ab (#70257, Cell Signaling) was diluted 1:100 in 1% bovine serum albumin (BSA) in PBS. Section slides were incubated with the primary antibody overnight at 4 °C, in humidified chamber. Following 2 washes in PBS, the section slides were incubated with the Cy-5 conjugated secondary antibody (similarly diluted in 1% BSA in PBS solution) for 1 hr at RT in the dark. Following 2 washes in PBS, the slides were mounted by fluorescent Mounting Medium with DAPI.

Slides were visualized using confocal microscopy (FV1200, Olympus, Japan), equipped with a 60×/1.42 oil immersion and 40×/0.95 air objective, images were scanned with 561 nm for dTomato, 488 nm for Dendra2, 405 nm for DAPI, 635 nm for Cy5 and transmitted light image. Images were analyzed using ImageJ software (National Institutes of Health, Bethesda, MD).

### Statistical analysis

Parametric or non-parametric tests were used based on the Shapiro-Wilk normality test. For comparison of two groups, two-tailed student’s *t*-test (for normally distributed data sets), Mann–Whitney (for unpaired non-normally distributed data sets) or Wilcoxon matched-pairs signed rank test (for paired non-normally distributed data) were used. For multiple comparison of three normally distributed data sets, one-way ANOVA followed by Dunnett’s or Tukey’s post-hoc tests was used. Kruskal–Wallis or Friedman tests with Dunn’s post-hoc tests were used for the analysis of non-normally distributed unpaired or paired data set, respectively. Detailed information on sample size and statistical tests used is described in figure legends. Data are presented as mean ± SEM. Differences between experimental groups were considered significant at *p < 0.05, **p < 0.01, ***p < 0.001, and ****p < 0.0001. *P* and t(df) values (for *t*-test), *U* (n_1_, n_2_) values (for Mann–Whitney test), F(DFn, DFd) values (for ordinary one-way ANOVA), or K–W or *F*-stat (for Kruskal–Wallis or Friedman tests, respectively), are stated in figure legends. Measurements were taken from distinct samples.

### Reporting summary

Further information on research design is available in the Nature Research Reporting Summary linked to this article.

## Supplementary information

Supplementary Information

Reporting summary

## Data Availability

mtDNA sequencing data produced in this study (aligned to the mitochondrial Cambridge Reference Sequence (CRS) [NC_012920.1]) have been submitted to the NCBI Sequence Read Archive (SRA; https://www.ncbi.nlm.nih.gov/sra/) under accession number PRJNA754853. Data supporting the findings of this paper are available from the corresponding authors upon reasonable request.

## Code availability

Short script for intensity profile (Supplementary Fig. 1) was upload to https://zenodo.org/record/5068427#.YOF18uhvaUk.
