# Supplementary material for: Mitochondrial augmentation of CD34+ cells from healthy donors and patients with mitochondrial DNA disorders confers functional benefit
Source: NPJ Regen Med. 2021 Sep 24;6:58. doi: 10.1038/s41536-021-00167-7 (PMC8463667; doi:10.1038/s41536-021-00167-7)
Supplement: Supplementary file 1 — Supplementary Information [file 41536_2021_167_MOESM1_ESM.pdf]

**Supplementary Materials:**

## Materials

**One primer set PCR method:** Mitochondrial DNA (mtDNA) was amplified in a 50 µl reaction containing 2.5 U of PrimeSTAR GXL DNA Polymerase Primer set based on a CLIA approved protocol[57,58] (mt16426F: 5'-CCGCACAAGAGTGCTACTCTCCTC, mt16425R: 5'-GATATTGATTTCACGGAGGATGGTG). Cycling was 98°C for 120 seconds and then 30 cycles of: 98°C for 10 seconds, 60°C for 15 seconds and 68°C for 170 seconds, followed by a 68°C hold for 50 seconds then hold at 12°C. Resulting amplicons were cleaned with 0.5X ratio SPRI Beads (AMPure XP, Beckman Coulter) analyzed by Agilent Tapestation on genomic DNA tape and sequenced as described in main text.

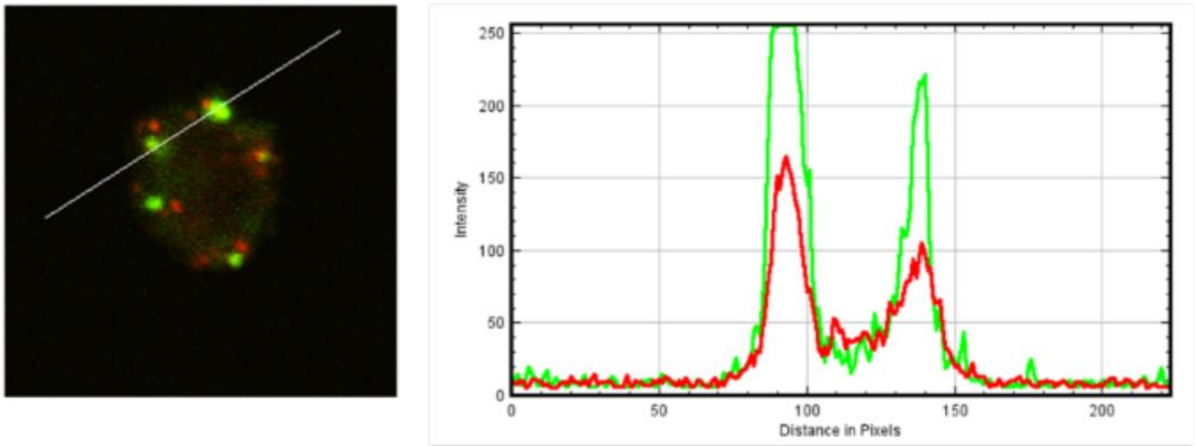


**Supplementary Figure 1: Exogenous and endogenous mitochondria can colocalize in augmented human $CD34^+$ cells.** Microscopy image of human $CD34^+$ stained with mitoTracker orange and subsequently augmented with GFP-labelled mitochondria isolated from HeLa cells 24 hr post augmentation. Colocalization is demonstrated using self-written macro [59] assessing correlated intensity peaks between the two fluorescent channels in the raw 2D image slices.

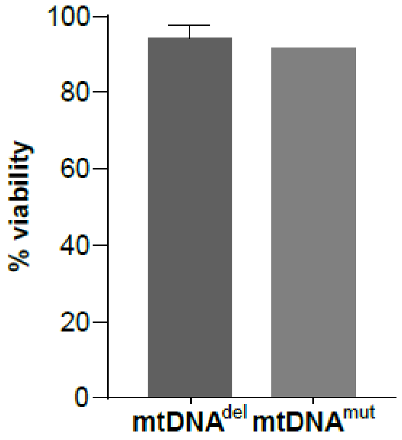


**Supplementary Figure 2: Mitochondrial augmentation of patient-derived CD34$^+$ with syngeneic mitochondria does not impair cell viability.** mtDNA$^{del}$ or mtDNA$^{mut}$ patient CD34$^+$ cells were augmented with syngeneic blood-derived mitochondria isolated from a blood unit donated by the patient's mother. Cell viability was tested 21 hr post augmentation (mtDNA$^{del}$ n=7 patients; mtDNA$^{mut}$ n=1 patient).

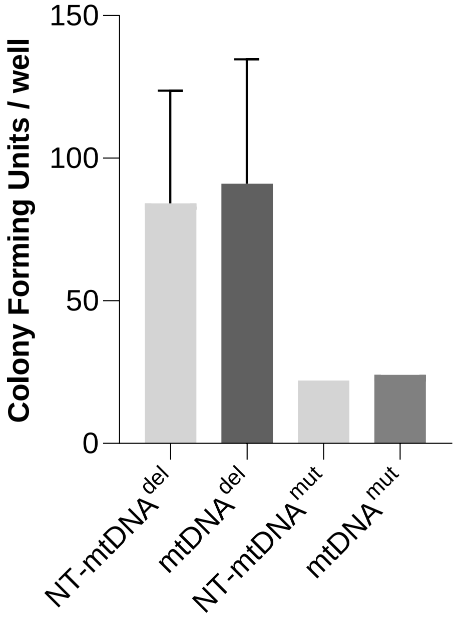


**Supplementary Figure 3: Mitochondrial augmentation of patient-derived CD34$^{+}$ with syngeneic mitochondria does not impair colony forming potential.** mtDNA$^{del}$ or mtDNA$^{mut}$ patient CD34$^{+}$ cells were augmented with syngeneic blood-derived mitochondria isolated from a blood unit donated by the patient's mother. The ability to form colonies in vitro was tested 14 days (+/- 2 days) post augmentation (mtDNA$^{del}$ n=10 patients; mtDNA$^{mut}$ n=1 patient).

**Gating strategy**

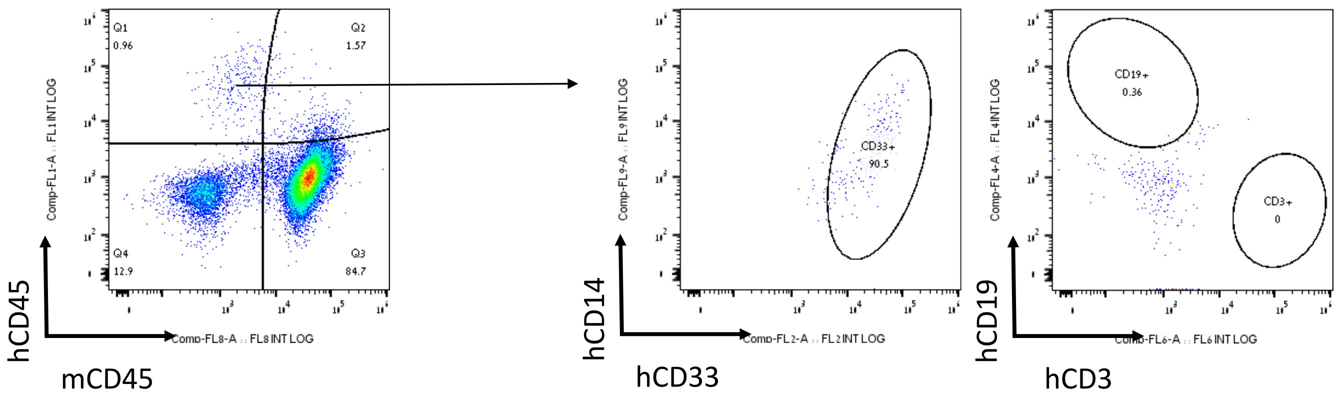


**Supplementary Figure 4: Flow cytometry gating strategy**. hCD45$^{+}$ cells were gated for CD33$^{+}$ cells, CD3$^{+}$ cells and CD19$^{+}$ cells (corresponds to Figure 3C).

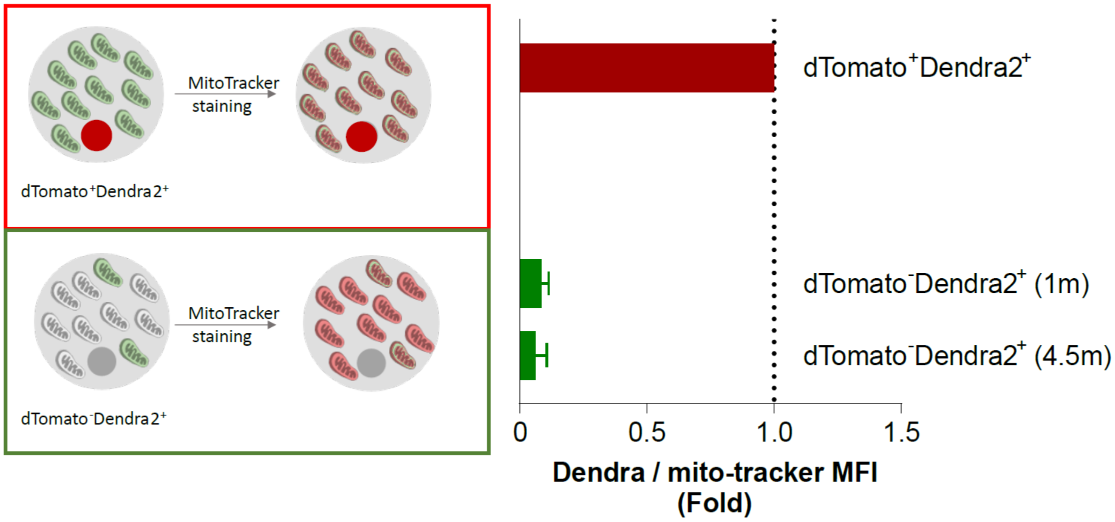


**Supplementary Figure 5: Exogenous mitochondria calculations.** Peripheral blood cells from the 1 and 4.5 month time points were stained with MitoTacker Deep-Red. All mitochondria are expected to be labelled with MitoTracker Deep-Red, whereas only exogenous mitochondria are expected to be labeled with Dendra2$^{+}$. The MFI of MitoTracker and Dendra2$^{+}$ was quantitated and the ratio of exogenous to total mitochondria was calculated. Based on the comparison between the Dendra2:MitoTracker Deep-Red ratio in dTomato$^{-}$Dendra2$^{+}$ cells vs. dTomato$^{+}$Dendra2$^{+}$ cells, we calculated that exogenous mitochondria were roughly 8.4% of total mitochondrial content in recipient cells (dTomato$^{-}$Dendra2$^{+}$) at 1 m and 6.1% at 4.5 m of the total cell mitochondria content. Importantly, this is a lower limit for exogenous mitochondria content, as it is limited by the half-life of the nuclear-encoded Cox8-Dendra2$^{+}$ protein which labels exogenous mitochondria.

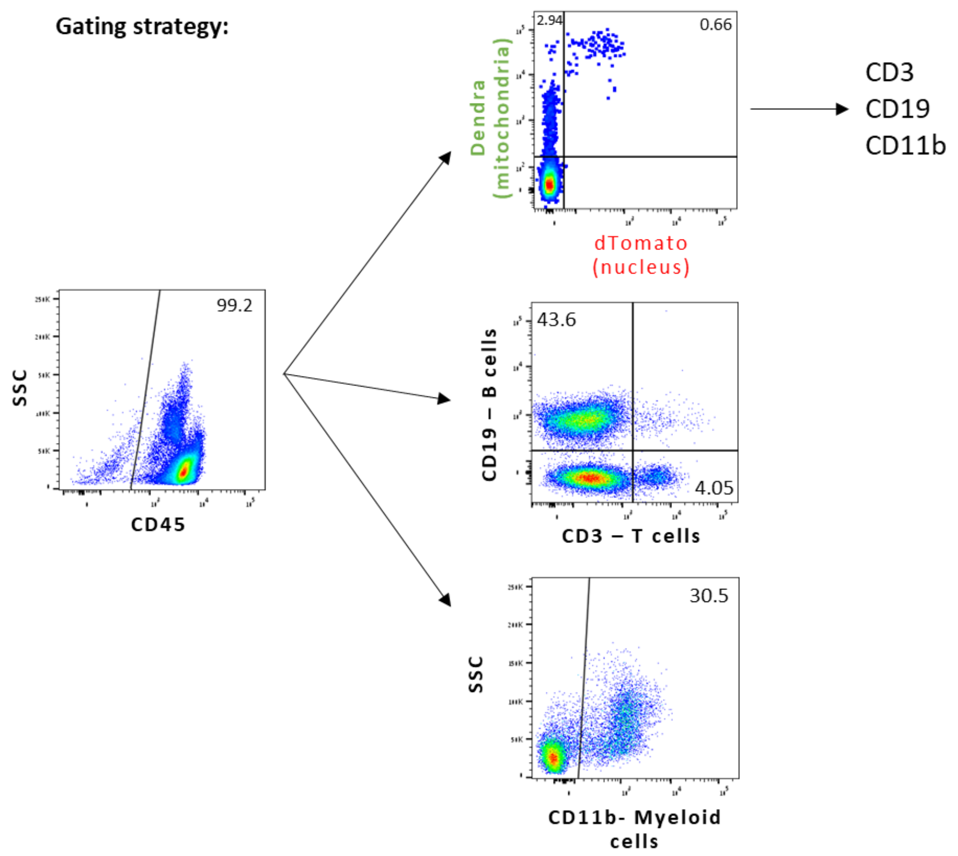


**Supplementary Figure 6: Flow cytometry gating strategy**. CD45$^+$ cells were gated for dTomato-Dendra2 cells, CD3$^+$ cells, CD19$^+$ cells and CD11b$^+$ cells (corresponds to Figure 4D).

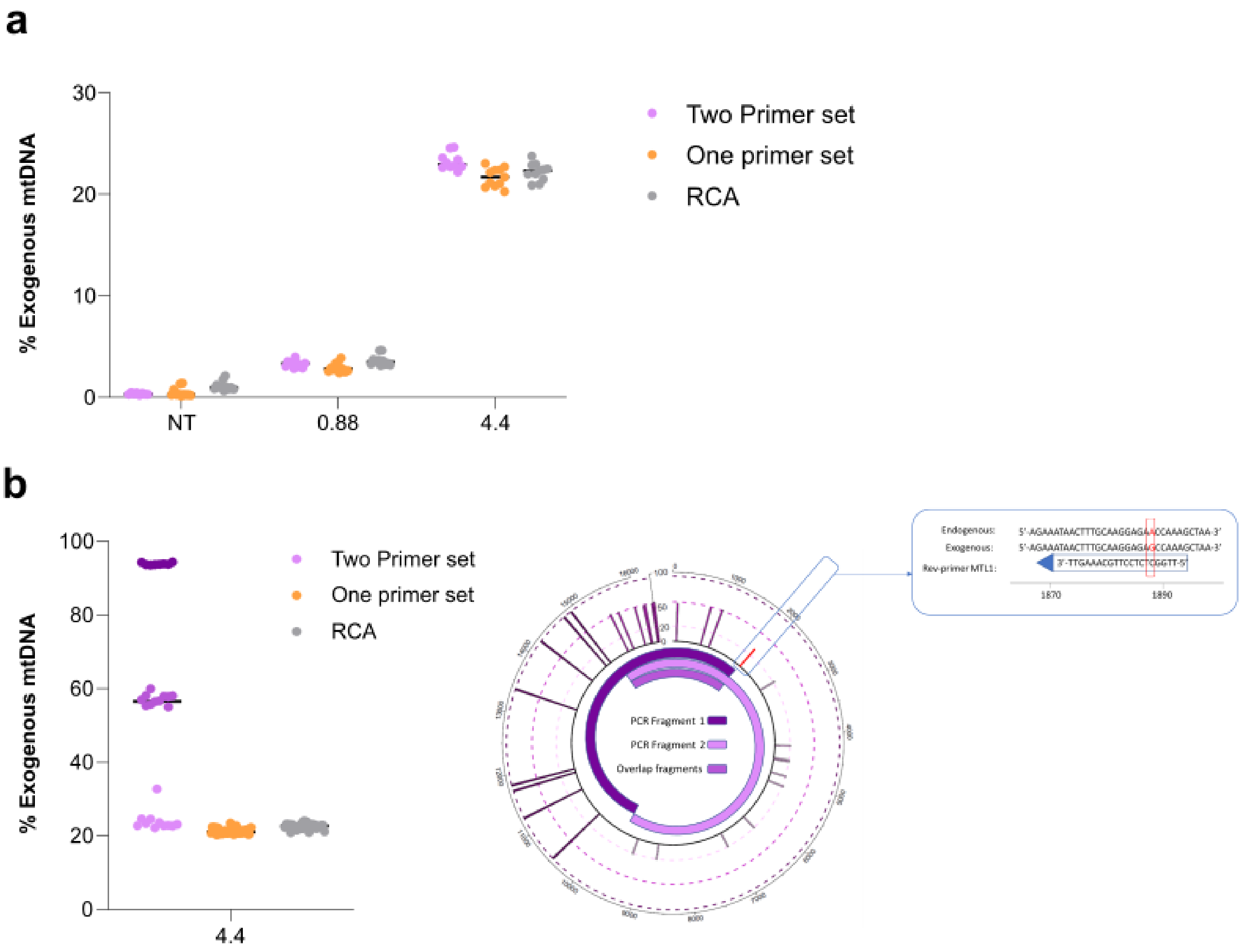


**Supplementary Figure 7: PCR and RCA based mtDNA sequencing based methodologies are comparable.**

(a) A comparison between several mtDNA sequencing methodology strategies (described in Methods section and one primer set method detailed below) performed on PBMCs (untreated, NT, or augmented at two doses, 0.88 or 4.4 mU CS) demonstrates similarity in the measured heteroplasmy levels irrespective of whether % exogenous mtDNA was calculated after mtDNA were enriched by PCR based on two primer sets (purple), a single PCR primer set (orange) and rolling cycle amplification (RCA)[60] (gray). (b) Demonstration of potential bias in PCR-based results due to primer set sensitivity to SNP specific to exogenous or endogenous mtDNA. An example of PCR bias that occurred due to a SNP at the annealing location of the MTL1 reverse primer that differentiated between endogenous and exogenous mtDNA, biasing the PCR step to select for exogenous mtDNA. Three different measurements of % exogenous mtDNA can be identified: all SNPs sequenced only by the MTL2 PCR fragments show an average of

23% augmentation, all SNPs sequenced by MTL1 PCR fragment show an average of 94% augmentation, and all SNPs covered by both fragments show an average of 57% augmentation. The actual % exogenous mtDNA is that as sequenced by PCR fragment MTL2, 23%, whose annealing primers do not differentiate between exogenous and endogenous mtDNA.  The % exogenous mtDNA as reported using the RCA-based mtDNA enrichment prior to sequencing, not expected to be significantly biased by annealing of a specific primer set, is consistent with this result (23% exogenous mtDNA).

## Supplementary references:


57. Luo, S. et al. Biparental inheritance of mitochondrial DNA in humans. *Proc. Natl. Acad. Sci. U. S. A.* **115**, 13039–13044 (2018).
58. Ma, H. et al. Metabolic rescue in pluripotent cells from patients with mtDNA disease. *Nature* **524**, 234–238 (2015).
59. Schindelin, J. et al. Fiji - an Open platform for biological image analysis. *Nat. Methods* **9**, (2012).
60. Marquis, J. et al. MitoRS, a method for high throughput, sensitive, and accurate detection of mitochondrial DNA heteroplasmy. *BMC Genomics* **18**, 1–19 (2017).
