# Supplementary material for: Mitochondrial augmentation of CD34+ cells from healthy donors and patients with mitochondrial DNA disorders confers functional benefit
Source: NPJ Regen Med. 2021 Sep 24;6:58. doi: 10.1038/s41536-021-00167-7 (PMC8463667; doi:10.1038/s41536-021-00167-7)
Supplement: Supplementary file 2 — Reporting summary [file 41536_2021_167_MOESM2_ESM.pdf]

nature portfolio

Corresponding author(s): Noa Sher, Elad Jacoby

Last updated by author(s): 2021 08 18

Reporting Summary

Nature Portfolio wishes to improve the reproducibility of the work that we publish. This form provides structure for consistency and transparency in reporting. For further information on Nature Portfolio policies, see our Editorial Policies and the Editorial Policy Checklist.

Statistics

For all statistical analyses, confirm that the following items are present in the figure legend, table legend, main text, or Methods section.

n/a	Confirmed	
☐	☒	The exact sample size (*n*) for each experimental group/condition, given as a discrete number and unit of measurement
☐	☒	A statement on whether measurements were taken from distinct samples or whether the same sample was measured repeatedly
☐	☒	The statistical test(s) used AND whether they are one- or two-sided<br>*Only common tests should be described solely by name; describe more complex techniques in the Methods section.*
☒	☐	A description of all covariates tested
☐	☒	A description of any assumptions or corrections, such as tests of normality and adjustment for multiple comparisons
☐	☒	A full description of the statistical parameters including central tendency (e.g. means) or other basic estimates (e.g. regression coefficient) AND variation (e.g. standard deviation) or associated estimates of uncertainty (e.g. confidence intervals)
☐	☒	For null hypothesis testing, the test statistic (e.g. *F*, *t*, *r*) with confidence intervals, effect sizes, degrees of freedom and *P* value noted<br>*Give P values as exact values whenever suitable.*
☒	☐	For Bayesian analysis, information on the choice of priors and Markov chain Monte Carlo settings
☒	☐	For hierarchical and complex designs, identification of the appropriate level for tests and full reporting of outcomes
☒	☐	Estimates of effect sizes (e.g. Cohen's *d*, Pearson's *r*), indicating how they were calculated

Our web collection on statistics for biologists contains articles on many of the points above.

Software and code

Policy information about availability of computer code

Data collection	n/a
Data analysis	Short script for intensity profile was upload to<br>https://zenodo.org/record/5068427#.YOF18uhvaUk<br>NGS data were analyzed by open source tools described in the materials section

For manuscripts utilizing custom algorithms or software that are central to the research but not yet described in published literature, software must be made available to editors and reviewers. We strongly encourage code deposition in a community repository (e.g. GitHub). See the Nature Portfolio guidelines for submitting code & software for further information.

Data

Policy information about availability of data

All manuscripts must include a data availability statement. This statement should provide the following information, where applicable:

- Accession codes, unique identifiers, or web links for publicly available datasets
- A description of any restrictions on data availability
- For clinical datasets or third party data, please ensure that the statement adheres to our policy

mtDNA sequencing data produced in this study (aligned to the mitochondrial Cambridge Reference Sequence (CRS) [NC_012920.1]) have been submitted to the NCBI Sequence Read Archive (SRA; https://www.ncbi.nlm.nih.gov/sra/) under accession number PRJNA754853.
Data supporting the findings of this paper are available from the corresponding authors upon reasonable request.

Field-specific reporting

Please select the one below that is the best fit for your research. If you are not sure, read the appropriate sections before making your selection.

☒ Life sciences ☐ Behavioural & social sciences ☐ Ecological, evolutionary & environmental sciences

For a reference copy of the document with all sections, see nature.com/documents/nr-reporting-summary-flat.pdf

Life sciences study design

All studies must disclose on these points even when the disclosure is negative.

Sample size	We did not use statistical methods to calculate sample sizes, however, our sample sizes are similar to those reported in related publications in the field.
Data exclusions	No data were excluded.
Replication	For in vitro studies with healthy donor's CD34+ cells, n=3 or n=4 was performed.<br>For patient cells, due to scarcity of the cells, one patient cell line was augmented with 2-4 mitochondrial batches.
Randomization	For in vitro studies, cells were randomized into groups before treatment. NSGS mice were randomized into two groups before cell injection. Randomization was not relevant to Polg mice model.
Blinding	Blinding was not relevant to in vitro studies as same investigator performed the experiment, data collection and analysis. Cell injection to NSGS mice was blinded.

Reporting for specific materials, systems and methods

We require information from authors about some types of materials, experimental systems and methods used in many studies. Here, indicate whether each material, system or method listed is relevant to your study. If you are not sure if a list item applies to your research, read the appropriate section before selecting a response.

Materials & experimental systems

n/a	Involved in the study	
☐	☒	Antibodies
☐	☒	Eukaryotic cell lines
☒	☐	Palaeontology and archaeology
☐	☒	Animals and other organisms
☐	☒	Human research participants
☒	☐	Clinical data
☒	☐	Dual use research of concern

Methods

n/a	Involved in the study	
☒	☐	ChIP-seq
☐	☒	Flow cytometry
☒	☐	MRI-based neuroimaging

Antibodies

Antibodies used	For NSGS mouse model<br>mCD45 – BioLegend, Cat. No. 103154, clone 30-F11, lot B330012<br>hCD45 – BioLegend, Cat. No. 304006, clone HI30, lot B293670<br>CD3 – BioLegend, Cat.No. 300412,clone UCHT1, lot B292782<br>CD33 – BioLegend, Cat. No. 366608 ,clone p67.6, lot B284832<br>CD19 – BioLegend, Cat. No. 363014, clone SJ25C1, lot B282678<br><br>For Polg mouse model<br>CD45- BioLegend, Cat. No. 103126, Clone 30-F11, Lot: B279412<br>CD19- BioLegend, Cat. No. 115531, Clone 6D5, Lot: B269485<br>CD11b- BioLegend, Cat. No. 101229, Clone M1/70, Lot: B256660<br>CD3- BioLegend, Cat. No. 100219, Clone 17A2, Lot: B284567
Validation	All antibodies have a Certificate Of Analysis stating that product lot has passed BioLegend's QC testing and is certified for use.

Eukaryotic cell lines

Policy information about cell lines

Cell line source(s)	CD34+ cells were purchased from stemexpress (Cat. No. MLEGP34010C) and Hemacare (Cat. No. M34C-GCSF/MOZ-3).
Authentication	Cells purity is specified by supplier as >= 90 % by flow cytometry.
Mycoplasma contamination	Testing for mycoplasma contamination was not relevant since cells were only grown for up to 24h.
Commonly misidentified lines (See ICLAC register)	n/a

Animals and other organisms

Policy information about studies involving animals; ARRIVE guidelines recommended for reporting animal research

Laboratory animals	For NSGS mice model, three weeks old female NSG-SGM3 (NSGS) mice (Jackson# 013062) were used. For Polg mice model, 24-27 weeks old males and 30-34 weeks old females Polg mice (Jackson# 017341) were used. As cell donors, 30-34 weeks old males and 31-34 weeks old females ROSAnT-nG/ PhAMexcised mice (generated by crossing Jackson stocks# 018397 & 023537) were used.
Wild animals	n/a
Field-collected samples	n/a
Ethics oversight	For NSGS mice model, protocols 1095/17/ANIM and 1252/20/ANIM were approved. For Polg mice model ethics commission approval number IL-19-3-129.

Note that full information on the approval of the study protocol must also be provided in the manuscript.

Human research participants

Policy information about studies involving human research participants

Population characteristics	Patients suffering from mitochondrial DNA deletions or mutations were treated under a clinical study (NCT03384420) or compassionate use program. Patient's age ranged from 3-18 years old.
Recruitment	Participants were under a clinical study (NCT03384420) or compassionate use program at Sheba medical center in Israel.
Ethics oversight	Study protocols were approved by Sheba medical center IRB and Israeli ministry of health.

Note that full information on the approval of the study protocol must also be provided in the manuscript.

Flow Cytometry

Plots

Confirm that:

- [x] The axis labels state the marker and fluorochrome used (e.g. CD4-FITC).
- [x] The axis scales are clearly visible. Include numbers along axes only for bottom left plot of group (a 'group' is an analysis of identical markers).
- [x] All plots are contour plots with outliers or pseudocolor plots.
- [x] A numerical value for number of cells or percentage (with statistics) is provided.

Methodology

Sample preparation	For NSGS mice model, Isolated bone marrow cells were incubated for 5 min with RBC lysis buffer, washed with PBS and stained for 30 min with different combinations of antibodies. Cells were then washed with PBS, and resuspended in FACS buffer prior to acquisition. For Polg mice model, Isolated bone marrow cells were stained for 25 min with different combinations of antibodies. Cells were then washed with PBS, fixed with 1% PFA for 10 min at 4°C and resuspended in FACS buffer prior to acquisition.
Instrument	For NSGS mice model, Gallios flow cytometer was used (BD biosciences). For Polg mice model, BD LSRFortessa flow cytometer (BD Biosciences) was used.
Software	FlowJo Software v10 was used for data analysis.
Cell population abundance	n/a

Gating strategy

For NSGS mouse model - the relevant cell population was gated according to FSC-SSC parameters. hCD45 stained cells were plotted against mCD45 cells. hCD45+ cells were gated for CD33+ cells, CD19+ cells and CD3+ cells.

☒ Tick this box to confirm that a figure exemplifying the gating strategy is provided in the Supplementary Information.
